# Neural Basis of Professional Pride in the Reaction to Uniform Wear

**DOI:** 10.3389/fnhum.2019.00253

**Published:** 2019-07-23

**Authors:** Yeon-Ju Hong, Sunyoung Park, Sunghyon Kyeong, Jae-Jin Kim

**Affiliations:** ^1^Department of Cognitive Science, Yonsei University, Seoul, South Korea; ^2^Institute of Behavioral Sciences in Medicine, Yonsei University College of Medicine, Seoul, South Korea; ^3^Department of Psychiatry, National Health Insurance Service Ilsan Hospital, Goyang, South Korea; ^4^Department of Psychiatry, Yonsei University College of Medicine, Seoul, South Korea

**Keywords:** professional pride, uniforms, reward network, emotion regulation, group cohesion

## Abstract

Professional pride is a positive emotion that includes self-reflection or evaluation and attitude toward one’s own occupational group. Uniforms can encourage the wearer to have professional pride. The current study aimed to elucidate the neural basis of professional pride using an experimental task related to the self in uniform and functional magnetic resonance imaging (fMRI). The person-adjective matching task, in which a participant or other in uniform or casual wear was presented with positive and negative words, was used for scanning fMRI. Imaging data from 21 adults who had an occupation requiring a uniform were analyzed to identify the main and interaction effects of individual (self vs. other), clothes (uniform vs. casual wear), and valence (positive vs. negative). Identified brain activities were correlated with psychological scales including the Rosenberg Self-esteem Scale and Group Environment Questionnaire. Whole brain analyses found that the interaction between individual and clothes was present in multiple regions such as the right ventrolateral prefrontal cortex (VLPFC), left dorsolateral prefrontal cortex, left middle and inferior temporal gyri, left posterior superior temporal sulcus, right temporoparietal junction, left lingual gyrus, left calcarine cortex, right insula, left caudate, and right putamen. In particular, activities in the right VLPFC, left calcarine cortex, and right putamen in the self/uniform condition were positively correlated with several psychological scales. These results suggest that professional pride may be represented through multiple brain networks related to empathy, reward, and emotion regulation as well as the theory-of-mind network. The neural basis of professional pride is closely related to positive self-evaluation and group cohesion.

## Introduction

Apparel has instrumental functions such as ensuring self-concept through clothing and enhancing self-esteem through other people’s positive responses to the meaning of the clothing (Solomon, 1983). The clothing represents the wearers themselves and is usually used as an “identity kit” to convey a desired impression to others (Scheff, 2005). By receiving or imagining a response from others, people perceive their appearance and achieve the idea of self that they are trying to define (Hormuth, 1990). In particular, the uniform, one of social apparel, is designed to act as a visual symbol to group members and can yield easier identification of roles as well as increased positivity (Adomaitis and Johnson, 2005). Wearing a uniform is also an effective means of identifying members and non-members and contributes to obtain social recognition from others or a sense of belonging (Lapitsky, 1961). Uniforms can give legitimate authority to certain roles in specific situations, promoting a sense of competence among the wearer and others. For example, wearing police uniforms gives the belief and authority that members will perform much more competently and responsibly than when they wear casual wear (Daniel, 1996). To sum up, uniforms can help foster a sense of pride and belonging and promote professionalism. In other words, uniforms can encourage the wearer to have a positive emotion of professional pride.

Pride is a self-focused emotion experienced when an individual or a group rises in social status (Tracy and Robins, 2007) and is a subjective, status-related, self-conscious emotion (Bolló et al., 2018). Expressing high status, which is beneficial for both the displayers and observers, is a social function of pride (Martens et al., 2012). Pride facilitates navigation in the social hierarchy and drives an individual to behave in socially appropriate ways (Tracy and Robins, 2004; Steckler and Tracy, 2014). Previous neuroimaging studies have reported that pride engages theory-of-mind (ToM)-related regions such as the medial prefrontal cortex (MPFC) and posterior superior temporal sulcus (pSTS) or temporo-parietal junction (TPJ) because it involves appraisals of social meaning (Takahashi et al., 2008) and self-referential processing (SRP)-related regions such as the MPFC and precuneus, as it is a self-oriented state (Zahn et al., 2009; Simon-Thomas et al., 2012; Roth et al., 2014).

Professional pride includes self-reflection or evaluation and attitude toward one’s own occupational group. With respect to the neural correlates of professional pride, it would engage ToM-related and SRP-related regions because of having pride as an element, while brain regions related to professionalism are also expected to be involved. Professionalism includes empathy, teamwork, and lifelong learning as key elements (San-Martín et al., 2017). It has been considered that professionalism may also include various other attributes such as the acceptance of a commitment to service, social responsibility and accountability, reliability, specialized knowledge, and self-regulation (LaSalal and Nelson, 2005). Although these elements cannot all be assumed to be related to professional pride, much is expected to be involved and possibly linked with the functions of different brain regions. For example, personal responsibility in decision-making produces a characteristic neurophysiological change (Li et al., 2011), and the empathy network including the anterior cingulate cortex (ACC) and insula (Engen and Singer, 2013) and teamwork-related brain reward responses (Morawetz et al., 2014) have been consistently reported. Therefore, in addition to the ToM-related and SRP-related regions, more brain regions would be involved in professional pride, but little is known about the neural correlates of this positive emotion.

In the current study, we drew inferences that uniforms can be effective visual means to examine the positive facet of professional pride, and thus developed a person-adjective matching task in which uniforms were used with casual wears as control stimuli and positive and negative words were used for assessing pride. The purpose of the study was to elucidate the neural basis of professional pride through functional magnetic resonance imaging (fMRI) using this task. We hypothesized that the positive emotion provoked by matching the self in uniform and pride-related words would recruit the empathy network or reward pathway as well as the ToM-related and SRP-related regions.

## Materials and Methods

### Participants

We recruited participants from the age of 25–40 years old who had a uniform-dressing job through Internet advertising. Among the volunteers, those with left-handedness as screened using the Edinburgh Handedness Inventory (Oldfield, 1971) and any neurological history or psychiatric illness were excluded. Finally, 22 volunteers participated in the experiment so as to include as many occupational groups as possible (six nurses, three soldiers, two medical doctors, two dentists, two radiological technologists, two bankers, one pilot, one stewardess, one athlete, one researcher, and one lawyer). However, because the data from one participant (one banker) who provided incomplete behavioral responses owing to drowsiness were discarded, the analysis only included data from the remaining 21 participants (10 females/11 males, mean age: 29.86 ± 4.14 years, age range: 25–39 years). This study was approved by the Institutional Review Board of Gangnam Severance Hospital, Yonsei University and carried out in accordance with the Declaration of Helsinki. All participants provided written informed consent before the start of the experiment.

### Self-Report Assessments

To investigate the psychological factors that could affect the positive self-evaluation, the Rosenberg Self-esteem Scale (RSES; Rosenberg, 1965) was assessed. To measure group cohesion, the Group Environment Questionnaire (GEQ; Carron et al., 1985) was administered and four subscale scores including Group Integration-Task, Group Integration-Social, Individual Attractions to Group-Task, and Individual Attractions to Group-Social were counted. Additionally, to measure the pride of wearing uniforms, we used our own questionnaire (the Uniform Questionnaire; UQ) to ask about the feel of uniforms. This contained three items, as follows: “When I wear the uniforms of our group, I feel responsible and think I should do better,” “When I meet someone else, I am proud and confident that I am wearing a uniform,” and “When I wear my uniform, I become more engaged and focused on my work.” Each question was scored according to a five-point Likert scale (from 1 = not at all to 5 = extremely), and thus total scores ranged from three to 15 points.

### Behavioral Task

**FIGURE 1:**
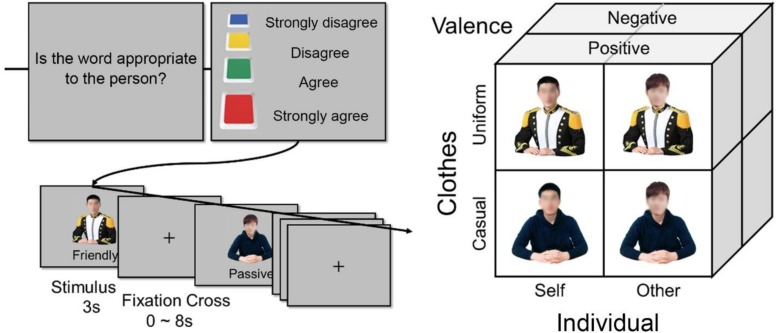
The person-adjective matching task, in which the images of self or other in uniform or casual wear are serially presented with positive or negative adjectives.

Before the fMRI experiment, participants were photographed to make their own pictures which were shown on the screen during the experiment. They were asked to bring in their own uniforms and casual wear to take pictures. The photographs were taken while looking at the front with a neutral facial expression and sitting in uniform or casual wear with both hands on the table, and were edited to show only the upper body. As shown in Figure 1, the picture conditions were prepared in four different ways: one’s own face and uniform, one’s own face and casual wear, the other’s face and one’s own uniform, and the other’s face and one’s own casual wear. The other’s face was that of a same-sex person unfamiliar to the participant and one of the neutral faces of three males or three females selected from the Korean Facial Expressions of Emotion (KOFEE; Park et al., 2011), which was edited to take only the face part to replace the participant’s face. As word stimuli, we used 10 positive trait adjectives (e.g., “generous”) and 10 matching negative trait adjectives (e.g., “unfriendly”), which were selected from a normalized pool (Anderson, 1968). The picture was edited to place the word under the person of the upper body. The backgrounds of the pictures were masked in gray color. Adobe Photoshop CS6 software (Adobe Systems Incorporated, San Jose, CA, United States) was used for editing the images.

Since there were 20 words in each of the four condition types, a total of 80 visual stimuli were produced in one set, in which positive and negative words were configured to match the same number in each of the self, other, uniform, and casual wear. Next, the other set of 80 visual stimuli was produced in a way such that the matching words were interchanged between the opposite conditions. During the fMRI experiment, the task sequence was separated into two sessions, in which the duration of each trial was 3 s and the inter-trial intervals varied from 0 to 8 s. Each session was configured to randomly present 80 events consisting of a set of pre-generated stimuli, and took a total duration of 8 min and 10 s. During the experiment, participants were asked to answer to a question of “Is the word appropriate to the person?” by pressing one of the four corresponding buttons for “strongly disagree,” “disagree,” “agree,” and “strongly agree.”

### Imaging Data Acquisition and Preprocessing

MRI data were acquired on a 3-Tesla scanner (Magnetom Verio; Siemens Medical Solutions, Erlangen, Germany). Functional images were collected using an echo planar sequence (echo time = 30 ms; repetition time = 2,000 ms; flip angle = 90°; slice thickness = 3 mm; field of view = 240 mm; and matrix = 64 × 64). T1-weighted images were also collected using a 3D spoiled-gradient-recall sequence (echo time = 2.46 ms; repetition time = 1,900 ms; flip angle = 9°; slice thickness = 1 mm; number of slices = 176; and matrix size = 256 × 256).

Using the Statistical Parametric Mapping (SPM)12 (Wellcome Department of Cognitive Neurology, Institute of Neurology, London, United Kingdom^1^) and MATLAB 2018a (Mathworks, Natick, MA, United States), the following image-preprocessing steps were conducted in order: realignment on the first image, slice-timing correction, co-registration and spatial normalization using a standard Montreal Neurological Institute (MNI) template, and smoothing using a Gaussian kernel with a full-width at half maximum of 8 mm.

### Behavioral Response Analysis

The agreement ratings were defined by assigning from −2 for “strongly disagree” to 2 for “strongly agree” in the positive word condition (the degree of agreement) and were defined as the inverse of the number assigned in the same way in the negative word condition (the degree of disagreement), so that higher scores indicated a positive view for both conditions. To determine the influence of individual (self vs. other), clothes (uniform vs. casual wear), and valence (positive vs. negative), repeated-measures analysis of variance (ANOVA) and a *post hoc* paired *t*-test were conducted on the positive ratings and response time (RT) using the Statistical Package for the Social Sciences (SPSS) version 17.0.0 software program.

### Imaging Data Analysis

Analysis was performed using a general linear model at the single-subject level. Images of the parameter estimates for different conditions were created during first-level analysis, during which individual realignment parameters were entered as regressors to control for the movement-related variance. There were eight conditions possible, from the combination of the two types of individual (self and other), two types of clothes (uniform and casual wear), and two types of valence (positive and negative), i.e., from self/uniform/positive to other/casual wear/negative. Second-level analysis was executed in a 2 (individual) × 2 (clothes) × 2 (valence) flexible repeated-measures ANOVA to identify brain regions showing the main and interaction effects. Statistical threshold was set at voxel-level *p* < 0.001 (uncorrected) at first, and then all clusters that met false discovery rate (FDR) corrected *p* < 0.05 at the cluster level were considered significant. Next, based on our hypothesis that the self/uniform condition would be associated with positive self-evaluation and pride in collective belonging, we extracted beta values in the self/uniform condition from the clusters showing significant interaction effects of individual × clothes using the Marsbar toolbox for SPM 12^2^. Their regional activity values were used to calculate the correlations with psychological assessment scores, such as the RSES score and four subscales scores of the GEQ. The significance level was *p* = 0.01 (0.05/5), considering that there were the correlations with the five scores.

## Results

### Behavioral Results

Participants reported the positive response to their uniform wearing; the mean total score of the UQ (range 3–15; median 9) were 10.71 ± 2.55. The UQ showed a good internal consistency, which was proven by Cronbach’s alpha of 0.88 based on participants’ responses. Supplementary Table 1 shows the results from the other self-report assessments. Behavioral responses in each condition during the experimental task are presented in Supplementary Table 2.

**FIGURE 2:**
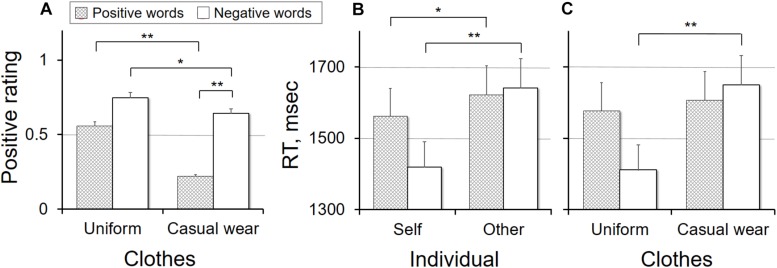
Behavioral findings showing the interaction effect. Significant results were identified in the positive rating between clothes and valence **(A)**, reaction time (RT) between individual and valence **(B)**, and RT between clothes and valence **(C)**. ^*^p < 0.05, ^∗∗^*p* < 0.01.

The agreement rating revealed significant main effects of individual, clothes, and valence. It was significantly higher in the self condition than in the other condition (0.74 ± 0.42 and 0.35 ± 0.50, respectively; *F*_(1,20)_ = 21.78, *p* < 0.001), in the uniform condition than in the casual condition (0.65 ± 0.44 and 0.43 ± 0.44, respectively; *F*_(1,20)_ = 14.19, *p* = 0.001), and for the negative words than for the positive words (0.69 ± 0.40 and 0.39 ± 0.52, respectively; *F*_(1,20)_ = 12.18, *p* = 0.002). A significant interaction was found only between clothes and valence (*F*_(1,20)_ = 7.65, *p* = 0.012); the agreement rating was significantly higher in the uniform condition than in the casual condition for both the positive words (0.56 ± 0.57 and 0.22 ± 0.55, respectively; *t*_20_ = 3.76, *p* = 0.001) and the negative words (0.75 ± 0.40 and 0.64 ± 0.44, respectively; *t*_20_ = 2.21, *p* = 0.045). However, conversely, it was significantly higher for the negative words than for the positive words in the casual condition (*t*_20_ = 4.13, *p* = 0.001), but not so in the uniform condition (*t*_20_ = 2.05, *p* = 0.053) (Figure 2A).

Response time showed significant main effects of individual and clothes, but no main effect of valence; it was significantly shorter in the self condition than in the other condition (1,490.77 ± 210.57 and 1,631.02 ± 215.08 ms, respectively; *F*_(1,20)_ = 12.53, *p* = 0.002) and in the uniform condition than in the casual condition (1,493.86 ± 219.48 and 1,627.93 ± 210.33 ms, respectively; *F*_(1,20)_ = 10.30, *p* = 0.004). A significant interaction was found only between individual and valence (*F*_(1,20)_ = 4.87, *p* = 0.039) and between clothes and valence (*F*_(1,20)_ = 5.65, *p* = 0.028). In *post hoc* analysis, participants responded significantly faster in the self condition than in the other condition for both the positive words (1,561.75 ± 220.15 and 1,621.38 ± 207.10 ms, respectively; *t*_20_ = −2.43, *p* = 0.025) and the negative words (1,419.78 ± 310.93 and 1,640.65 ± 229.08 ms, respectively; *t*_20_ = 3.06, *p* = 0.006). However, the difference between the self and other conditions was significantly greater for the negative words than for the positive words (220.87 ± 330.77 and 59.63 ± 112.36 ms, respectively; *t*_20_ = 2.21, *p* = 0.039) (Figure 2B). Furthermore, participants’ responses were significantly faster in the uniform condition than in the casual condition for the negative words (1,411.07 ± 340.31 and 1,649.36 ± 226.79 ms, respectively; *t*_20_ = 2.86, *p* = 0.010), but there was no difference between the two conditions for the positive words (Figure 2C).

### Imaging Results

#### Brain Regions Related to the Factors

**FIGURE 3:**
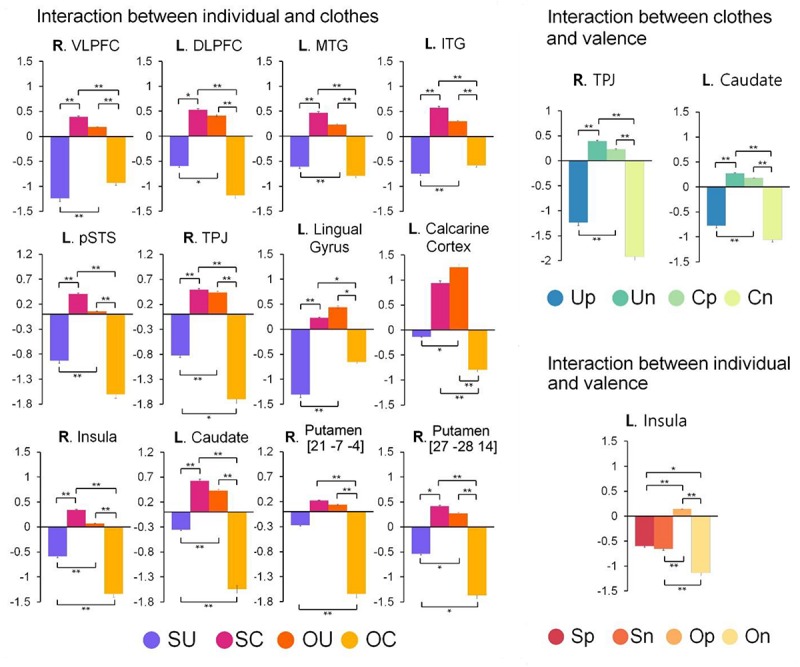
Comparison of regional activity among the conditions in the regions showing the interaction effects. The numbers under the subtitles for the right putamen mean the Montreal Neurological Institute (MNI) coordinates. SU, the self/uniform condition; SC, the self/casual condition; OU, the other/uniform condition; OC, the other/casual condition; Up, the uniform/positive condition; Un, the uniform/negative condition; Cp, the casual/positive condition; Cn, the casual/negative condition; Sp, the self/positive condition; Sn, the self/negative condition; Op, the other/positive condition; and On, the other/negative condition; R., right; L., left; VLPFC, ventrolateral prefrontal cortex; DLPFC, dorsolateral prefrontal cortex; MTG, middle temporal gyrus; ITG, inferior temporal gyrus; pSTS, posterior superior temporal sulcus; TPJ, temporoparietal junction. ^*^p < 0.05, ^∗∗^*p* < 0.01.

As shown in Table 1, whole-brain analysis yielded significant main effects of individual, clothes, and valence. The main effect of individual was found in the bilateral frontopolar cortex, right ventrolateral prefrontal cortex (VLPFC), and right supramarginal gyrus. In *post hoc* tests, all of these regions showed increased activity in the self condition compared to the other condition. Brain regions showing the main effect of clothes were the right VLPFC and right putamen, where activity was increased in the uniform condition compared to the casual condition. The main effect of valences was identified in the right ACC, right VLPFC, bilateral supramarginal gyrus, and bilateral insula, all of which showed increased activity in the positive condition compared to the negative condition. Whole-brain analysis also yielded significant interaction effects of individual × clothes, clothes × valence, and individual × valence, but there was no interaction of individual × clothes × valence. The interaction between individual and clothes was found in multiple regions such as the right VLPFC, left DLPFC, left middle and inferior temporal gyri, left pSTS, right TPJ, left lingual gyrus, left calcarine cortex, right insula, left caudate, and right putamen. The interaction between clothes and valence was observed in the right TPJ and left caudate, and the interaction between individual and valence was identified only in the left insula. Differences among the conditions in the regions showing the interaction effects are presented in Figure 3.

**TABLE 1:** The clusters showing the significant main and interaction effects.

	**MNI coordinate**				**MNI coordinate**		
**Main effect Brain region (BA)**	***x/y/z***	**Number voxels**	***Z*_max_**	**Interaction effect Brain region (BA)**	***x/y/z***	**Number voxels**	***Z*_max_**
**Individual**				**Individual × Clothes**			
L. FPC (10)	−48/47/−4	49	4.09	R. VLPFC (47)	24/29/5	58	4.80
R. FPC (10)	45/44/−7	34	3.86	L. DLPFC (8)	−30/14/47	35	4.30
R. VLPFC (45)	54/20/5	109	4.67	L. MTG (21)	−42/−49/2	51	3.73
R. SMG (40)	39/−55/56	29	3.52	L. ITG (21)	−57/−7/−22	57	4.62
				L. pSTS (39)	−39/−55/26	127	4.43
**Clothes**				R. TPJ (39)	39/−67/29	312	5.54
R. VLPFC (44)	45/11/20	85	4.09	L. Lingual gyrus (19)	−18/−52/8	30	4.23
R. Putamen	21/−10/2	40	5.06	L. Calcarine cortex (17)	−12/−91/5	36	3.63
				R. Insula (13)	30/11/23	147	4.70
**Valence**				L. Caudate	−2/−13/23	280	5.05
R. ACC (32)	3/26/38	79	4.34	R. Putamen	21/−7/−4	51	3.88
R. VLPFC (44)	57/17/8	153	4.58		27/−28/14	138	4.06
R. SMG (40)	42/−34/41	103	4.35	**Clothes × Valence**			
L. SMG (40)	−48/−37/38	132	4.72	R. TPJ (39)	39/−73/32	55	4.89
L. Insula (13)	−33/17/−1	70	3.98	L. Caudate	−21/−13/20	23	4.22
R. Insula (13)	42/2/8	50	4.52	**Individual × Valence**			
				L. Insula (13)	−33/−16/26	103	4.97

*Significant clusters were obtained at voxel-level *p*_*unc*_ < 0.001 and cluster-level *p*_*FDR*_ < 0.05. BA, Brodmann area; MNI, Montreal Neurological Institute; L., left; R., right; FPC, frontopolar cortex; VLPFC, ventrolateral prefrontal cortex; SMG, supramarginal gyrus; ACC, anterior cingulate cortex; DLPFC, dorsolateral prefrontal cortex; ITG, inferior temporal gyrus; MTG, middle temporal gyrus; pSTS, posterior superior temporal gyrus; TPJ, temporoparietal junction.*

#### Correlations Between Brain Activity and Behavioral Variables

**FIGURE 4:**
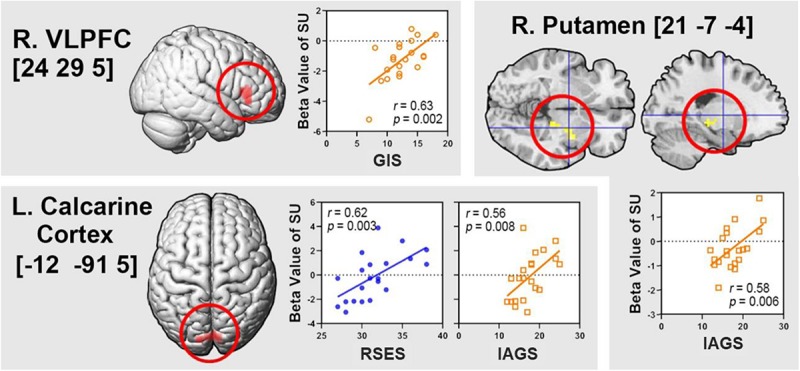
Brain regions showing the interaction effect between individual and clothes, and significant correlations of regional activity in the self/uniform condition (SU) with behavioral variables. The numbers associated with the region name mean the Montreal Neurological Institute (MNI) coordinates. R., right; L., left; VLPFC, ventrolateral prefrontal cortex; RSES, Rosenberg Self-esteem Scale; GIS, Group Integration-Social in the Group Environment Questionnaire (GEQ); IAGS, Individual Attractions to Group-Social in the GEQ.

Among the brain regions showing the interaction effect between individual and clothes, significant correlations between regional activity in the self/uniform condition and behavioral variables were found in three regions such as the right VLPFC, left calcarine cortex, and right putamen (Figure 4), as follows: right VLPFC activity with the level of Group Integration-Social in the GEQ (*r* = 0.63, *p* = 0.002); left calcarine cortex activity with the RSES scores (*r* = 0.62, *p* = 0.003) and the level of Individual Attractions to Group-Social in the GEQ (*r* = 0.56, *p* = 0.008); and right putamen activity with the level of Individual Attractions to Group-Social in the GEQ (*r* = 0.58, *p* = 0.006).

## Discussion

This study was performed to elucidate the neural basis of professional pride in the reaction to uniform wear using the person-adjective matching task. In the behavioral results, participants reported a positive response in the self-report regarding their uniform wear and their agreement ratings in the uniform condition were higher in comparison with the casual condition, suggesting that uniform wear induces positive emotions. Particularly, because the agreement ratings were higher in the self condition than in the other condition, these positive emotions are likely to be related to professional pride. The certainty of this view is supported by the other behavioral findings reflecting the confidence of participants, in that the degree of disagreement with the negative words was greater than that of agreement with the positive words, and this disagreement with the negative words was faster in the self condition than in the other condition and in the uniform condition than in the casual condition.

Our main hypothesis included the involvement of the ToM-related and SRP-related regions in professional pride. Previous neuroimaging studies have shown that several distinct regions form an integrated functional network for ToM reasoning, and the MPFC in the anterior brain and the pSTS and TPJ in the posterior brain form the core in the network (Carrington and Bailey, 2009). The MPFC is a critical region for mentalizing, whereas the pSTS and TPJ play an important role in perspective-taking (Frith, 2007). In our experiment, these ToM-related regions were found in the posterior brain, but not in the anterior brain. A previous study suggested a functional dissociation within the ToM network for different mental contents, with a common recruitment for cognitive and affective states in the pSTS and TPJ, but not in the MPFC (Corradi-Dell’Acqua et al., 2014). The only involvement of these posterior regions was also reported in a previous study of pride, which did not find the engagement of the MPFC, a region responsible for self-reflection, probably because pride might require less self-reflection compared to negative self-conscious emotions such as guilt or embarrassment (Takahashi et al., 2008). Likewise, the reason for why the SRP-related regions such as the MPFC and precuneus (Northoff et al., 2006; Uddin et al., 2007) did not appear in the results may be that professional pride is relatively less self-reflective. Meanwhile, a neural effect of linear combination of the self and uniform factors would be expected in our experiment. However, the ToM-related regions in the self/uniform condition showed greater activation relative to the other/causal condition, but not relative to the self/causal and other/uniform conditions. This results suggest that ToM-related neural activity in the self/uniform condition may be a product of complicated non-linear rather than linear combination of the self and uniform factors involving the self-evaluation and group cohesion processes.

Our additional hypothesis was that professional pride would recruit the empathy network and reward pathway. The imaging results showing the interaction of individual and clothes included the insula, which is part of the empathy network (Engen and Singer, 2013) and is also involved in emotion regulation and reward processing (Tanaka et al., 2004; Villafuerte et al., 2012). It has been known that the insula uses secondary reward signals and integrates contingencies to compensate for the negative feeling of social pain (Cristofori et al., 2015). However, it is unlikely that insula activity is confined to negative feelings alone. Our task provoked a positive feeling rather than a negative feeling, as shown in the behavioral results. It should be noted that the insula is activated when an individual is faced with choices that have both positive and negative social outcomes (Knutson and Greer, 2008).

Other evidence of the involvement of the reward pathway in professional pride is that the interaction of individual and clothes was found in the striatum such as the caudate and putamen. It is well-known that the striatum plays a critical role in processing both monetary and social rewards (Izuma et al., 2008; Albrecht et al., 2014). This role has been confirmed by some previous findings of enhanced caudate activity in response to recalling positive autobiographical memories (Speer et al., 2014) and reward-augmenting reciprocated cooperation (Rilling et al., 2012). In addition, our results showed that putamen activity in the self/uniform condition was positively correlated with the level of individual attractions to group-social. The function of the putamen supported by this finding is consistent with the role of the striatum that integrates social information into the coding of social action and reward (Báez-Mendoza and Schultz, 2013). The role of the striatum is likely to include cooperating behaviors in that this region is activated while working together to complete a maze (Krill and Platek, 2012). Considering that empathy and teamwork are key elements of professionalism (San-Martín et al., 2017), our findings on the insula and striatum support an important role of the empathy and reward networks in professional pride.

In our study, activity in the lateral prefrontal regions such as the VLPFC and DLPFC also showed the interaction of individual and clothes. Accumulative data have suggested that these two regions are involved in numerous higher cognitive processes including working memory, implementation of top-down goals and plans, episodic retrieval, inhibition, and self-control (Snow, 2016), and have dissociable roles; for example, the VLPFC may implement action control, whereas the DLPFC may represent the task goal (Swann et al., 2013). Furthermore, previous neuroimaging studies have found that both the VLPFC and DLPFC are core regions involved in various kinds of emotion regulation (Buhle et al., 2014; Kohn et al., 2014). While the DLPFC plays a general role in emotion regulation, reflecting a cognitive demand for regulation (Golkar et al., 2012), the VLPFC is engaged in both the generation and regulation of emotion through subcortical pathways including the striatum and amygdala (Wager et al., 2008). These two regions are both implicated in processing of social hierarchy (Zink et al., 2008; Marsh et al., 2009). A previous study reported that both regions are activated while viewing social interaction video clips related to dominance, suggesting their roles in power-related social motivations (Quirin et al., 2013). Looking at the more specialized features, the VLPFC has been demonstrated in the regulation of social exclusion and the reduction of social pain (Eisenberger et al., 2003; He et al., 2018). Based on these previous reports, our finding that VLPFC activity in the self/uniform condition was positively correlated with the level of group integration-social may suggest the role of this region in the generation and regulation of emotion in a social context. This role is certainly important in that uniform wear leads to emotional affirmation as a member of the organization and also to the need for behavioral abstinence. Taken together, the involvement of the VLPFC and DLPFC in self and uniform processing may reflect professionalism-related social responsibility and self-regulation.

There are some limitations in the current study. First, because of the small sample size, sex or career variation was not analyzed. Second, in some cases, there may be occupations where uniform wear is negative rather than positive, and these groups were not included in the current study. Third, the connectivity issue was not determined even though we discussed various networks based on the activated regions. Therefore, future research with effective connectivity analysis is required to address this issue.

## Conclusion

The current study using the person-adjective matching task and fMRI for elucidating the neural basis of professional pride revealed that brain activity related to the self in uniform was found in various regions including the VLPFC, DLPFC, pSTS, TPJ, insula, and striatum. These results suggest that professional pride may be represented through multiple brain networks related to empathy, reward, and emotion regulation as well as the ToM network. These findings may reflect the characteristics of uniform wear including emotional affirmation as a member of the organization and the need for behavioral abstinence. Therefore, the neural basis of professional pride is closely related to positive self-evaluation and group cohesion.

## Ethics Statement

This study was approved by the Institutional Review Board of Gangnam Severance Hospital, Yonsei University and carried out in accordance with the Declaration of Helsinki. All participants provided the written informed consent before the start of the experiment.

## Author Contributions

SP and J-JK designed the study. Y-JH and SP acquired the data. Y-JH and SK analyzed the data. Y-JH and J-JK wrote the manuscript. All authors reviewed and approved the final manuscript.

## Conflict of Interest Statement

The authors declare that the research was conducted in the absence of any commercial or financial relationships that could be construed as a potential conflict of interest.

## Footnotes

1 http://www.fil.ion.ucl.ac.uk/spm

2 http://marsbar.sourceforge.net
