# Supplementary material for: Neural Basis of Professional Pride in the Reaction to Uniform Wear
Source: Front Hum Neurosci. 2019 Jul 23;13:253. doi: 10.3389/fnhum.2019.00253 (PMC6664020; doi:10.3389/fnhum.2019.00253)
Supplement: Supplementary file 1 [file Table_1.DOC]

| **Supplementary Table 1.** The results of self-report assessments | | | | | |
| --- | --- | --- | --- | --- | --- |
| Conditions | Minimum | Maximum | Mean | Standard deviation |  |
| Rosenberg Self-esteem Scale | 27 | 38 | 31.48 | 3.19 |  |
| Group Environment Questionnaire |  |  |  |  |  |
| Group Integration-Task | 11 | 23 | 16.57 | 2.71 |  |
| Group Integration-Social | 7 | 18 | 12.43 | 2.79 |  |
| Individual Attractions to Group-Task | 10 | 20 | 15.00 | 2.45 |  |
| Individual Attractions to Group-Social | 12 | 25 | 17.52 | 3.71 |  |
| Uniform Questionnaire | 6 | 15 | 10.71 | 2.55 |  |
