# Supplementary material for: Neural Basis of Professional Pride in the Reaction to Uniform Wear
Source: Front Hum Neurosci. 2019 Jul 23;13:253. doi: 10.3389/fnhum.2019.00253 (PMC6664020; doi:10.3389/fnhum.2019.00253)
Supplement: Supplementary file 2 [file Table_2.DOC]

| **Supplementary Table 2**. Mean and standard deviation of the positive rating and response time (msec) in each condition. | | | |
| --- | --- | --- | --- |
| Condition | | Mean | SD |
| Positive rating | |  |  |
|  | Self, Uniform, and Positive | 0.80 | 0.55 |
|  | Self, Uniform, and Negative | 0.90 | 0.43 |
|  | Self, Casual, and Positive | 0.46 | 0.67 |
|  | Self, Casual, and Negative | 0.78 | 0.41 |
|  | Other, Uniform, and Positive | 0.31 | 0.71 |
|  | Other, Uniform, and Negative | 0.60 | 0.49 |
|  | Other, Casual, and Positive | -0.02 | 0.53 |
|  | Other, Casual, and Negative | 0.50 | 0.56 |
| Response time | |  |  |
|  | Self, Uniform, and Positive | 1,542.04 | 219.92 |
|  | Self, Uniform, and Negative | 1,240.99 | 643.04 |
|  | Self, Casual, and Positive | 1,581.46 | 233.80 |
|  | Self, Casual, and Negative | 1,598.58 | 237.99 |
|  | Other, Uniform, and Positive | 1,611.24 | 235.58 |
|  | Other, Uniform, and Negative | 1,581.16 | 234.59 |
|  | Other, Casual, and Positive | 1,631.53 | 192.80 |
|  | Other, Casual, and Negative | 1,700.15 | 232.06 |
